# Using the Hybrid Community Care Model to Examine Implementation Domains in Rural Behavioral Health: Exploratory Pilot Study

**DOI:** 10.2196/102681

**Published:** 2026-08-25

**Authors:** David A Wilkerson, John M Keesler, Kristin Funk

**Affiliations:** 1 School of Social Work, Indiana University, ES Bldg 4138, 902 W. New York St., Indianapolis, IN, 46202, United States, 1 317-274-6705

**Keywords:** hybrid behavioral health, implementation science, rural health, care coordination, digital health, telehealth, community-based support, digital health equity

## Abstract

**Background:**

Rural communities continue to experience behavioral health disparities associated with workforce shortages, digital exclusion, and fragmented coordination between trusted community-based supports and formal behavioral health systems. Although telehealth has expanded opportunities for care, less is known about how key implementation factors interact within hybrid behavioral health systems—coordinated systems that integrate trusted community-based support with formal digital behavioral health services—or how these interactions influence implementation and engagement across community and formal care settings.

**Objective:**

This exploratory pilot study examined implementation domains relevant to hybrid behavioral health systems in rural communities using the hybrid community care model (HCCM) as the conceptual framework for survey development and interpretation of the findings.

**Methods:**

A cross-sectional survey was administered to professionals and community stakeholders attending a rural community workshop in the Midwestern United States, with a 46.3% (38/82) response rate. Survey items operationalized 4 theoretically informed implementation domains: relational trust, digital system capacity, coordination, and perceived feasibility of hybrid care models. Descriptive statistics and Spearman rank-order analyses were conducted to examine preliminary associations among individual survey items.

**Results:**

Spearman rank-order correlations indicated that coordination-related items demonstrated the strongest and most consistent associations, particularly among referral knowledge, collaboration between the community and behavioral health providers (BHPs), and continuity of care (ρ=0.383-0.633; *P*≤.02). Perceived hybrid care feasibility demonstrated more variable associations, with respondents perceiving more limited BHP and appointment availability reporting stronger support for community-BHP partnerships (ρ=−0.471 and −0.648, respectively; *P*=.004 and *P*<.001).

**Conclusions:**

Preliminary findings suggest that coordination warrants further investigation as an implementation process within hybrid behavioral health systems. The HCCM served as a conceptual framework for interpreting the observed perception patterns and provides a foundation for future hypothesis-driven implementation research. Additional studies using validated measures are needed to further evaluate the implementation domains represented within the HCCM and examine their applicability across diverse rural settings.

## Introduction

### Background

Rural behavioral health disparities persist across the United States, reflecting intersecting economic, structural, sociocultural, and geographic barriers that limit access to care and contribute to poorer outcomes across the lifespan [1-3]. These disparities arise from interacting conditions across multiple domains, including biological, behavioral, environmental, sociocultural, and health system factors [4-6], as well as emerging forms of digital exclusion that constrain equitable access to telehealth and other technology-enabled services [7,8].

Trusted community institutions in rural America, particularly churches and faith communities, often function as informal care networks that provide relational support yet may lack systematic connections to formal behavioral health and digital health systems [7,8]. At the same time, digital platforms and telehealth services have expanded the reach of professional expertise and hold promise for improving access in underserved areas [5,7]. However, disparities in broadband access, digital literacy, affordability, and trust continue to limit their reach [1,5,6,9], and coordination with trusted local networks remains fragmented [10,11]. Consequently, trusted community actors, such as faith leaders, community organizations, and informal support networks, often coexist with available telehealth services in rural communities; however, persistent digital exclusion and coordination gaps limit individuals’ ability to access, navigate, and sustain engagement with behavioral health care [7,12,13].

### Prior Work

Existing scholarship has largely examined community-based trust, telehealth expansion, and digital access independently [5,10,11]. Less is known about how these factors interact within hybrid behavioral health systems—coordinated systems that integrate trusted community-based support with formal behavioral health and digital health services. Existing implementation and systems frameworks, including the Consolidated Framework for Implementation Research (CFIR), recognize that implementation is shaped by interacting contextual, organizational, individual, and process-level determinants and identify coordination as an important implementation process influencing service delivery [14,15]. Studies of behavioral health integration similarly demonstrate that effective coordination depends on both structural mechanisms, such as referral pathways and shared workflows, and relational processes such as communication, trust, and collaboration across organizations [16,17]. These approaches have primarily focused on coordination within or between health care organizations and formal service systems, including primary care, behavioral health, and Veterans Affairs–community partnerships [18].

### Study Significance

In contrast, comparatively less attention has been given to conceptualizing coordination as the implementation process that integrates trusted community-based support systems with formal digital behavioral health services within hybrid behavioral health systems [4,9]. This represents the primary conceptual contribution of the hybrid community care model (HCCM), which extends existing implementation science perspectives by emphasizing coordination across community, digital, and formal behavioral health systems rather than within health care organizations alone [9].

**Figure 1.**
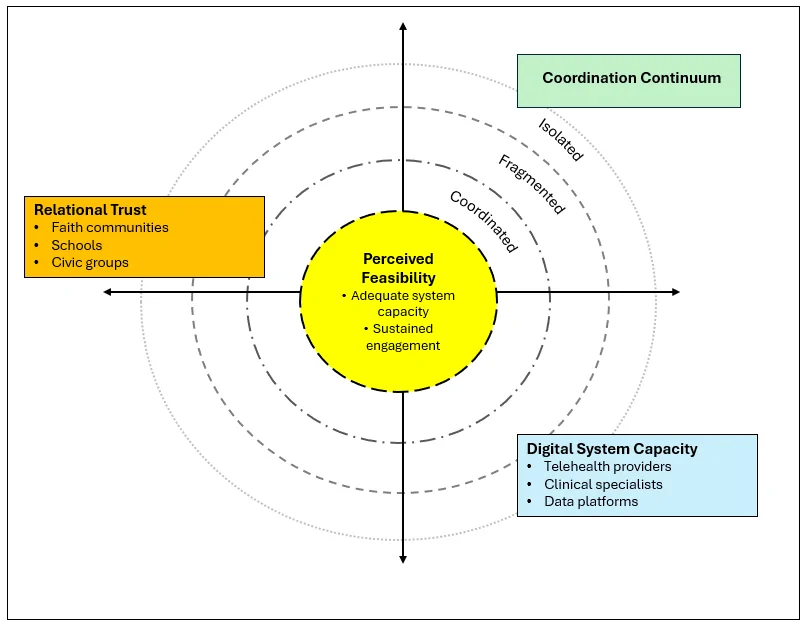
Hybrid community care model (HCCM): conceptual framework informing the study. HCCM conceptualizes how relational trust and digital system capacity interact through coordination to influence the perceived feasibility of hybrid behavioral health implementation in rural communities. The coordination continuum illustrates increasing integration between trusted community-based support systems and formal digital behavioral health services, progressing from isolation to coordinated collaboration.

To examine coordination processes, the HCCM (Figure 1) was developed through an iterative process integrating evidence from the rural behavioral health and implementation science literature with insights gained through community-based telehealth implementation in rural settings [19]. Early implementation efforts suggested that improving digital access alone did not ensure adoption of telebehavioral health services, highlighting the importance of relational trust, cross-sector coordination, and contextual feasibility as key implementation determinants [19,20]. These insights complemented the literature and informed the development of the HCCM, which served as the conceptual framework for this study.

Together, the theoretical and practice-based insights were synthesized into 4 interrelated domains representing the conditions that support hybrid behavioral health implementation. *Relational trust* refers to confidence in trusted community-based individuals and organizations that facilitate help seeking and engagement with behavioral health care [11,21]. *Digital system capacity* reflects the availability and readiness of technological, organizational, and workforce resources that support technology-enabled behavioral health services [4,9]. *Coordination* refers to the implementation processes, including referral pathways, collaboration, and continuity of care, that functionally connect trusted community-based support systems with formal digital behavioral health services [17,22]. *Perceived feasibility* of hybrid care models reflects judgments regarding the practicality, usefulness, and local implementation potential of integrating community-based support with formal digital behavioral health services [23,24].

### Study Aim

The aim of this exploratory pilot study was to examine associations among the 4 implementation domains in rural behavioral health settings. The HCCM informed survey development and interpretation of the findings as a framework for understanding implementation processes within hybrid behavioral health systems.

## Methods

### Study Design

This study used an exploratory, cross-sectional pilot survey design to examine preliminary associations among theoretically informed implementation domains relevant to hybrid behavioral health systems. Pilot studies are intended to assess the feasibility of research approaches, refine conceptual models, and generate preliminary evidence to inform larger, hypothesis-driven investigations rather than provide definitive tests of theory or intervention effectiveness [25,26]. An exploratory design was selected because the relationships among the proposed implementation domains have not previously been examined within a single conceptual framework. Exploratory research is particularly appropriate when investigating emerging phenomena and examining relationships among variables drawn from multiple theoretical perspectives before confirmatory testing [27,28]. A cross-sectional survey was used because it provides an efficient approach for examining associations among variables measured at a single point in time and is well suited to exploratory investigations of implementation-related factors [29].

The 4 implementation domains represent conceptually distinct but potentially interacting influences within hybrid behavioral health systems and guided survey development and interpretation of the findings. Similar theory-informed approaches have been used to examine relationships among implementation domains in health care settings before formal theory testing [30].

Consistent with the exploratory purpose of the study, analyses focused on identifying preliminary associations among the domains rather than testing causal hypotheses, establishing psychometric properties, or validating the HCCM as a measurement model. Findings are therefore interpreted as preliminary empirical evidence intended to inform subsequent hypothesis-driven research, implementation studies, and refinement of HCCM-informed implementation measures.

### Setting, Participants, and Recruitment

Data were collected during a regional professional development workshop for professionals working within rural community support systems, which was held in a rural county in the Midwestern United States in April 2026. The workshop focused on community well-being, population-level data, and strategies for strengthening services for children, youth, and families. Participants represented a broad range of family-serving organizations and community systems, including education, behavioral health, social services, public health, and other community-based organizations.

A purposive convenience sample was recruited from workshop attendees because the study sought perspectives from professionals and community stakeholders across rural community, health, education, and behavioral health systems. During the workshop introductions, the presenter made an oral announcement inviting all attendees to participate in an anonymous electronic survey.

Of the 82 workshop attendees, 38 completed the survey, yielding a response rate of 46.3% (38/82). Because the study was designed as an exploratory pilot investigation, the sample was intended to provide preliminary perspectives from professionals and community stakeholders engaged in rural service systems rather than to constitute a representative sample of rural residents.

### Survey Development

The study was reported in accordance with CHERRIES (Checklist for Reporting Results of Internet E-Surveys) [31]. A completed CHERRIES is provided in Checklist 1.

The survey was developed by the authors, who are social work researchers and educators, and informed by a synthesis of the rural behavioral health, telehealth, and implementation science literature and approximately 3 years of experience designing and implementing a rural public library telehealth initiative [19]. This initiative’s performance suggested that improving digital access alone did not ensure community participation. Although rural public library–based telehealth addressed digital access barriers, community uptake remained limited, inferring that relational trust, behavioral health stigma, and coordination between community organizations and behavioral health providers (BHPs) were also important implementation considerations. These performance insights informed the development of survey items representing the 4 implementation domains of the HCCM: relational trust, digital system capacity, coordination, and perceived feasibility of hybrid behavioral health care.

The authors independently drafted candidate items for each domain and refined them through iterative discussion until consensus was reached regarding item clarity, relevance, and alignment with the conceptual framework. The survey was intentionally designed as a brief exploratory instrument suitable for completion during a professional development workshop. The instrument was technically pretested by the authors before administration to verify item functionality, navigation, and electronic data capture within Qualtrics.

### Measures

The electronic survey consisted of 12 substantive items representing the 4 implementation domains described previously, together with demographic questions characterizing the study sample. To balance conceptual coverage with respondent burden during this exploratory pilot study, 3 conceptually distinct items were developed for each implementation domain. All substantive items used a 5-point Likert response scale ranging from 1 (“strongly disagree”) to 5 (“strongly agree”). The complete questionnaire is provided in Multimedia Appendix 1.

### Data Collection

Data were collected during and immediately following the workshop. Participants accessed the anonymous electronic survey by scanning a QR code displayed during the workshop. To maximize participation, a follow-up email containing the survey link was distributed to all workshop attendees, and the survey remained open for an additional 24 hours. Most responses were submitted during the workshop, with 2 additional responses received following the email reminder.

The survey was administered electronically using the Qualtrics XM survey platform (Qualtrics LLC) and required approximately 5 to 10 minutes to complete. Participation was voluntary, and respondents could decline participation or skip individual survey items, resulting in limited item-level missingness. No personally identifying information was collected, and responses were stored within the secure Qualtrics environment before export for analysis.

### Data Analysis

Descriptive statistics were calculated to summarize participant characteristics and response patterns for individual survey items. Because survey responses were measured using ordinal Likert scales, item-level responses were summarized using medians and IQRs. Spearman rank-order correlation coefficients were calculated to examine associations among individual survey items. Given the exploratory nature of the study, analyses focused on item-level relationships rather than aggregated domain scores.

The final analytic sample included 38 participants. Participants were permitted to skip individual survey questions; therefore, item-level missingness resulted in 92.1% (35/38) complete cases across all survey items. One survey response containing no substantive data was excluded before analysis. Pairwise deletion was used to maximize the available data for each correlation analysis, resulting in slight variation in sample size across individual correlations.

All analyses were conducted using SPSS (version 31.0; IBM Corp). Given the exploratory pilot design and modest sample size, the analyses were intended to identify moderate-to-strong associations among survey items rather than detect small effect sizes. Accordingly, the findings are interpreted as preliminary patterns of association intended to inform subsequent hypothesis-driven implementation domain studies rather than establish causal relationships.

### Ethical Considerations

This study was reviewed by the Indiana University Institutional Review Board (IRB) and determined to qualify for exempt review because it involved minimal-risk survey research with adult participants. Because it was exempt from ongoing IRB review, no protocol number was assigned. All study procedures were conducted in accordance with the ethics principles of the Declaration of Helsinki and its subsequent amendments.

Before accessing the survey, participants were presented with an electronic informed consent statement describing the purpose of the study, the voluntary nature of participation, and their right to decline participation or discontinue the survey at any time without penalty. Completion and submission of the survey indicated informed consent.

To protect participant privacy and confidentiality, no personally identifying information was collected. Survey responses were anonymous and stored electronically using the Qualtrics XM survey platform before export for analysis. Participants did not receive financial compensation or other incentives for completing the survey.

## Results

### Participant Characteristics

Of the 38 participants, most respondents identified as female (36/38, 94.7%). Educational attainment was relatively high, with the largest proportion reporting a graduate degree (13/38, 34.2%), followed by a bachelor’s degree (11/38, 28.9%). Smaller proportions reported a high school diploma or General Educational Development (GED; 6/38, 15.8%), some college education (4/38, 10.5%), or an associate’s degree (4/38, 10.5%). The response rate for demographic items varied slightly because participants could skip individual survey questions.

Participants represented a range of age groups. Approximately half of respondents were aged 38 to 47 years (19/37, 51.4%). Respondents aged 18 to 37 years comprised 18.9% (7/37), those aged 48 to 57 years comprised 16.2% (6/37), and those aged ≥58 years comprised 13.5% (5/37). Most respondents described the communities they served as rural (36/38, 94.7%), with the remainder identifying the communities they served as suburban (2/38, 5.3%).

### Descriptive Statistics

Descriptive statistics for all survey items are presented in Table 1. Respondents generally reported moderate-to-high levels of relational trust, limited BHP and appointment availability, moderate perceptions of coordination across community and formal behavioral health systems, and favorable perceptions of hybrid community-BHP partnerships.

**Table 1.** Descriptive statistics for survey items representing the 4 implementation domains (N=38).

Domains and survey items^a^	Valid responses per item, n (%)	Scores, median (IQR)	
Relational trust (Q1-3)			
Comfort in seeking help	37 (97.4)	4 (2-4)	
Trust in local groups	37 (97.4)	3 (3-4)	
Confidence in confidentiality	36 (94.7)	3 (2-4)	
Digital system capacity (Q4-6)			
BHP^b^ availability	36 (94.7)	1 (1-2)	
Appointment availability	35 (92.1)	2 (1-2)	
Telehealth availability	37 (97.4)	4 (3-4)	
Coordination (Q7-9)			
Referral knowledge	37 (97.4)	3 (2-4)	
Community-BHP collaboration	37 (97.4)	3 (2-3)	
Continuity across systems	37 (97.4)	3 (2-3)	
Perceived feasibility (Q10-12)			
Long-term partnership possible	37 (97.4)	4 (3-4)	
Hybrid care improves access	36 (94.7)	4 (4-4)	
Community-BHP partnerships useful	37 (97.4)	4 (4-5)	

a Item-level sample sizes vary because participants could skip individual survey questions.

b BHP: behavioral health provider.

Median responses indicated low perceived BHP availability (median 1, IQR 1-2) and appointment availability (median 2, IQR 1-2), whereas respondents generally agreed that hybrid care could improve access (median 4, IQR 4-4) and that community-BHP partnerships were useful (median 4, IQR 4-5). Response dispersion varied across survey items, with comfort in seeking help (median 4, IQR 2-4), confidence in confidentiality (median 3, IQR 2-4), and referral knowledge (median 3, IQR 2-4) demonstrating the greatest variability, whereas perceptions that hybrid care could improve access were concentrated at “agree” within the middle 50% of responses (median 4, IQR 4-4).

### Correlation Analyses

Table 2 presents the complete Spearman rank-order correlation analysis (ρ and 2-tailed *P* values) for all survey items. An abbreviated survey item description is provided for each item to facilitate interpretation. Correlation analyses identified several significant associations among survey items representing the 4 implementation domains.

**Table 2.** Spearman rank-order correlation analysis (ρ and 2-tailed *P* values) among survey items^a^.

Survey items	Q1	Q2	Q3	Q4	Q5	Q6	Q7	Q8	Q9	Q10	Q11	Q12
Comfort in seeking help (Q1)												
ρ	1	−0.097	0.416^b^	−0.024	0.032	−0.102	0.106	0.051	−0.013	0.093	−0.327	0.019
*P* value	―^c^	.57	.01	.89	.86	.55	.53	.76	.94	.59	.05	.91
Sample size (n)	37	37	36	36	35	37	37	37	37	37	36	37
Trust in local groups (Q2)												
ρ	−0.097	1	−0.099	0.115	0.134	0.295	0.297	0.09	−0.046	0.113	0.205	0.064
*P* value	.57	―	.57	.50	.44	.08	.07	.60	.79	.51	.23	.71
Sample size (n)	37	37	36	36	35	37	37	37	37	37	36	37
Confidence in confidentiality (Q3)												
ρ	0.416^b^	−0.099	1	−0.055	−0.093	−0.125	0.067	0.168	0.048	0.15	0.14	0.033
*P* value	.01	.57	―	.75	.60	.47	.70	.33	.78	.38	.42	.85
Sample size (n)	36	36	36	35	34	36	36	36	36	36	35	36
Behavioral health provider availability (Q4)												
ρ	−0.024	0.115	−0.055	1	0.565^d^	0.15	0.15	0.368^b^	0.676^d^	0.089	−0.239	−0.471^d^
*P* value	.89	.50	.75	―	<.001	.38	.38	.03	<.001	.61	.16	.004
Sample size (n)	36	36	35	36	35	36	36	36	36	36	36	36
Appointment availability (Q5)												
ρ	0.032	0.134	−0.093	0.565^d^	1	0.289	0.246	0.357^b^	0.493^d^	−0.02	−0.315	−0.648^d^
*P* value	.86	.44	.60	<.001	―	.09	.15	.04	.003	.91	.06	<.001
Sample size (n)	35	35	34	35	35	35	35	35	35	35	35	35
Telehealth availability (Q6)												
ρ	−0.102	0.295	−0.125	0.15	0.289	1	0.228	0.385^b^	0.079	0.341^b^	−0.037	−0.191
*P* value	.55	.08	.47	.38	.09	―	.18	.02	.64	.04	.83	.26
Sample size (n)	37	37	36	36	35	37	37	37	37	37	36	37
Referral knowledge (Q7)												
ρ	0.106	0.297	0.067	0.15	0.246	0.228	1	0.529^d^	0.383^b^	−0.226	0.042	−0.104
*P* value	.53	.07	.70	.38	.15	.18	―	<.001	.02	.18	.81	.54
Sample size (n)	37	37	36	36	35	37	37	37	37	37	36	37
Community–behavioral health provider collaboration (Q8)												
ρ	0.051	0.09	0.168	0.368^b^	0.357^b^	0.385^b^	0.529^d^	1	0.633^d^	0.161	−0.046	−0.218
*P* value	.76	.60	.33	.03	.04	.02	<.001	―	<.001	.34	.79	.20
Sample size (n)	37	37	36	36	35	37	37	37	37	37	36	37
Continuity of care (Q9)												
ρ	−0.013	−0.046	0.048	0.676^d^	0.493^d^	0.079	0.383^b^	0.633^d^	1	0.001	−0.3	−0.428^d^
*P* value	.94	.79	.78	<.001	.003	.64	.02	<.001	―	.99	.08	.008
Sample size (n)	37	37	36	36	35	37	37	37	37	37	36	37
Long-term partnership availability (Q10)												
ρ	0.093	0.113	0.15	0.089	−0.02	0.341^b^	−0.226	0.161	0.001	1	−0.068	0.154
*P* value	.59	.51	.38	.61	.91	.04	.18	.34	.99	―	.69	.36
Sample size (n)	37	37	36	36	35	37	37	37	37	37	36	37
Hybrid care improves access (Q11)												
ρ	−0.327	0.205	0.14	−0.239	−0.315	−0.037	0.042	−0.046	−0.3	−0.068	1	0.543^d^
*P* value	.05	.23	.42	.16	.06	.83	.81	.79	.08	.69	―	<.001
Sample size (n)	36	36	35	36	35	36	36	36	36	36	36	36
Community-provider partnerships useful (Q12)												
ρ	0.019	0.064	0.033	−0.471^d^	−0.648^d^	−0.191	−0.104	−0.218	−0.428^d^	0.154	0.543^d^	1
*P* value	.91	.71	.85	.004	<.001	.26	.54	.20	.008	.36	<.001	―
Sample size (n)	37	37	36	36	35	37	37	37	37	37	36	37

a Cells report Spearman rank-order correlation coefficients (ρ) and corresponding 2-tailed *P* values. Pairwise deletion was used because participants could skip individual survey items; therefore, sample sizes varied slightly across correlations (n=34-37). One response containing no substantive survey data was excluded before analysis. Because the analyses were exploratory, no adjustment was made for multiple comparisons.

b Correlation is significant at the .05 level (2-tailed).

c Not applicable.

d Correlation is significant at the .01 level (2-tailed).

Indicators of relational trust demonstrated relatively few significant associations outside the trust domain. Comfort in seeking help from trusted community organizations was positively associated with confidence in local groups maintaining confidentiality (ρ=0.416; *P*=.01), suggesting internal consistency among trust-related perceptions. Few additional associations were observed with indicators of digital system capacity, coordination, or perceived feasibility.

The strongest and most consistent associations were observed among coordination-related indicators. Referral knowledge, community-BHP collaboration, and continuity of care were all positively associated (ρ=0.383-0.633; *P*≤.02), indicating that these implementation processes tended to cluster together.

Indicators of digital system capacity demonstrated selective associations with coordination-related indicators. Greater perceived availability of BHPs, appointments, and telehealth were each associated with stronger perceptions of community-BHP collaboration and continuity of care, whereas referral knowledge showed comparatively limited relationships. These associations indicate that digital system capacity may support implementation primarily through enhanced coordination rather than greater awareness of referral pathways.

Indicators representing perceived feasibility of hybrid care models demonstrated more variable relationships. Respondents perceiving more limited BHP and appointment availability reported stronger support for community-BHP partnerships, whereas agreement that hybrid care could improve access was positively associated with perceived partnership usefulness. Overall, perceptions of hybrid care feasibility appeared to vary according to existing community and service conditions rather than increasing uniformly with higher levels of trust or digital system capacity.

## Discussion

### Principal Findings

This exploratory pilot study examined associations among respondents’ perceptions of 4 implementation domains relevant to hybrid behavioral health systems: relational trust, digital system capacity, coordination, and perceived feasibility of hybrid care models. Because the survey measured perceptions rather than objective implementation metrics, the findings should be interpreted as reflecting perceived implementation conditions within rural behavioral health systems.

Three principal findings emerged. First, respondents’ perceptions of coordination-related processes demonstrated the strongest and most consistent pattern of associations, suggesting that referral knowledge, collaboration between community-based and BHPs, and continuity of care are closely interconnected implementation processes within hybrid behavioral health systems. Second, perceptions of relational trust demonstrated comparatively few associations outside the trust domain. Although respondents generally reported favorable perceptions of trust, greater variability in comfort in seeking help and confidence in confidentiality suggests that trust-related perceptions were not uniform across respondents. Together, these findings suggest that relational trust may represent a foundational community condition rather than a direct indicator of system coordination. Finally, perceptions of hybrid care feasibility varied according to respondents’ perceptions of local service conditions, with those perceiving more limited BHP and appointment availability also expressing stronger support for community-BHP partnerships.

Collectively, these findings suggest that coordination warrants continued investigation as a distinct implementation process linking trusted community-based support systems with formal digital behavioral health services. Although this exploratory pilot study was not designed to test or validate the HCCM, the observed perception patterns are consistent with its conceptualization of coordination as the process through which community, digital, and clinical resources become aligned and provide direction for next steps in implementation research.

### Comparison With Prior Literature

Previous research consistently identifies relational trust as an important facilitator of help seeking and community engagement within rural behavioral health systems, particularly where trusted community organizations serve as initial points of contact for individuals experiencing behavioral health concerns [11,21,23]. The present findings complement this literature by suggesting that trust alone may not be sufficient to support effective coordination between community-based and formal behavioral health services. Instead, the observed variability in comfort in seeking help and confidence in confidentiality highlights that favorable perceptions of trust do not necessarily translate into uniform readiness for coordinated implementation across respondents.

Prior implementation studies demonstrate that effective coordination depends on communication, referral pathways, clearly defined roles, and collaborative relationships that connect organizations across service settings [16-18,21,22]. These findings extend this literature by suggesting that respondents perceived referral knowledge, collaboration, and continuity of care as closely interconnected implementation processes within hybrid behavioral health systems. This pattern suggests that respondents viewed coordination as a set of interdependent implementation activities rather than as isolated organizational functions, reinforcing the importance of coordination in connecting community-based and formal behavioral health services.

Implementation studies have shown that workforce shortages, infrastructure limitations, reimbursement uncertainty, and inadequate digital navigation influence perceptions regarding the practicality of hybrid behavioral health models [2-4,24]. Consistent with this literature, respondents perceiving more limited BHP and appointment availability also expressed stronger support for community-BHP partnerships, suggesting that perceived feasibility may reflect implementation need as much as existing system readiness. Together, these findings suggest that perceived feasibility may be shaped not only by available resources but also by respondents’ assessments of unmet implementation needs within their local communities.

### Implications for Hybrid Behavioral Health Implementation

The observed perception patterns suggest that implementation efforts in rural behavioral health may benefit from focusing on coordination processes in addition to expanding workforce capacity or digital infrastructure. Although increasing access to telehealth and other digital resources remains important, the present findings suggest that their perceived value may depend on how effectively they are integrated with existing community relationships and formal behavioral health services. Strengthening referral pathways, clarifying organizational roles, supporting digital navigation, and improving communication across organizations may therefore represent actionable implementation strategies for reducing fragmentation and improving continuity of care.

These implications extend across multiple stakeholder groups. For BHPs and health systems, the findings emphasize the importance of developing reliable coordination processes that connect clinical services with trusted community organizations. For community partners, including faith communities, schools, youth-serving organizations, and other locally trusted institutions, the findings suggest opportunities to strengthen engagement by serving as community anchors that facilitate help seeking, referral, and digital navigation while remaining connected to formal behavioral health systems. For rural communities, implementation efforts that build upon existing relational assets while strengthening coordination may represent a more feasible and sustainable approach than relying exclusively on expanding clinical services or technological resources.

From an implementation perspective, the HCCM provides a framework for considering how trusted community-based support systems, digital system capacity, and formal behavioral health services may be intentionally aligned rather than developed independently. Rather than viewing relational trust or technology as sufficient to improve access, the HCCM suggests that coordination is the implementation process through which these existing assets can be translated into meaningful engagement with care. Future implementation studies should therefore examine strategies that strengthen communication, referral pathways, role clarity, feedback mechanisms, and digital navigation across community and clinical settings while evaluating their effects on access, engagement, and continuity of care.

As an exploratory pilot study, the present investigation represents an initial step in operationalizing the implementation domains of the HCCM rather than validating the HCCM or establishing the psychometric properties of the survey instrument. Subsequent research should first establish the instrument’s psychometric properties using larger and more diverse samples, including exploratory and confirmatory factor analyses, assessments of internal consistency and test-retest reliability, and evaluations of construct validity. Once these measurement properties have been established, the instrument can be applied in larger implementation studies designed to examine the HCCM across diverse rural settings.

### Limitations

Several limitations should be considered when interpreting these findings. First, the study used a relatively small purposive convenience sample drawn from participants attending a professional development workshop in a single rural community. Consequently, the findings may not be generalizable to other rural populations or behavioral health systems. The modest sample size also limited the study’s ability to detect small associations; therefore, the analyses were primarily sensitive to moderate-to-strong relationships, and nonsignificant findings should be interpreted cautiously because weaker associations may not have been detected. Future studies using larger and more diverse samples are needed to evaluate smaller effect sizes and confirm the observed correlation patterns.

Second, pairwise deletion was used to maximize the available data for each correlation analysis, resulting in slight variation in sample size across correlations (n=34-37). Consequently, individual coefficients were estimated from slightly different subsets of participants, which may have introduced minor variability into the observed associations. Future studies with more complete data should evaluate these relationships using a consistent analytic sample.

Third, the cross-sectional design limits interpretation to associations observed at a single point in time and does not permit causal inference or examination of changes in implementation conditions over time. Longitudinal implementation research will be needed to examine how these relationships evolve across different settings and stages of implementation.

Finally, the survey was developed as an exploratory pilot instrument to operationalize the implementation domains of the HCCM rather than as a validated measurement scale. Although informed by implementation science, the rural behavioral health literature, and community-based telehealth implementation experience, the instrument has not yet undergone formal psychometric evaluation.

### Conclusions

This exploratory pilot study provides preliminary empirical evidence suggesting that respondents’ perceptions indicate that coordination may represent an important implementation process within hybrid rural behavioral health systems. Among the implementation domains examined, coordination-related indicators demonstrated the strongest and most consistent pattern of associations, indicating that respondents perceived referral knowledge, collaboration, and continuity of care as closely interconnected aspects of implementation.

Although the study was not designed to validate the HCCM, the observed perception patterns are consistent with its conceptualization of coordination as the process through which trusted community-based support systems and formal digital behavioral health services become aligned. More broadly, the HCCM provides a framework for examining how community relationships, digital system capacity, coordination, and perceived feasibility interact to influence implementation within rural behavioral health systems.

As rural communities continue to address persistent behavioral health workforce shortages and unequal access to care, implementation efforts that strengthen coordination between trusted community organizations and formal behavioral health services may support more integrated approaches to service delivery. Subsequent studies should evaluate these implementation domains using larger and more diverse samples, validated measures, longitudinal designs, and implementation-focused methodologies to further examine the HCCM and its application across diverse rural settings.

## Supplementary material

10.2196/102681 Multimedia Appendix 1 Survey instrument operationalizing the 4 hybrid community care model implementation domains: relational trust, digital system capacity, coordination, and perceived feasibility of hybrid care models.

10.2196/102681 Checklist 1 Completed CHERRIES checklist.

## Abbreviations

BHP: behavioral health provider
CFIR: Consolidated Framework for Implementation Research
CHERRIES: Checklist for Reporting Results of Internet E-Surveys
HCCM: hybrid community care model
IRB: Institutional Review Board
