# Supplementary material for: Using the Hybrid Community Care Model to Examine Implementation Domains in Rural Behavioral Health: Exploratory Pilot Study
Source: JMIR Form Res. 2026 Aug 25;10:e102681. doi: 10.2196/102681 (PMC13505968; doi:10.2196/102681)
Supplement: Supplementary file 1 [file formative-v10-e102681-s001.docx]

HCCM Survey

You are invited to participate in a brief research survey examining factors that influence behavioral health services in rural communities. Participation is voluntary. Your responses are anonymous, and no personally identifying information will be collected. You may skip any question or stop the survey at any time. Completion of the survey indicates your consent to participate.

Q1 People in my community feel comfortable asking for help from trusted local groups (like churches, schools, or community organizations).

- Strongly disagree (1)
- Disagree (2)
- Not sure (3)
- Agree (4)
- Strongly agree (5)

Q2 Leaders (like pastors or teachers) in my community are good first people to talk to about mental health concerns.

- Strongly disagree (1)
- Disagree (2)
- Not sure (3)
- Agree (4)
- Strongly agree (5)

Q3 People in my community trust local groups to keep personal information private.

- Strongly disagree (1)
- Disagree (2)
- Not sure (3)
- Agree (4)
- Strongly agree (5)

Q4 There are enough mental health providers in or near my community.

- Strongly disagree (1)
- Disagree (2)
- Not sure (3)
- Agree (4)
- Strongly agree (5)

Q5 People in my community can usually get a mental health appointment without long wait times.

- Strongly disagree (1)
- Disagree (2)
- Not sure (3)
- Somewhat agree (4)
- Strongly agree (5)

Q6 Online mental health services (like telehealth) are available for people in my community.

- Strongly disagree (1)
- Disagree (2)
- Not sure (3)
- Agree (4)
- Strongly agree (5)

Q7 In my community, local helpers (like pastors or teachers) know how to connect people with mental health professionals.

- Strongly disagree (1)
- Disagree (2)
- Not sure (3)
- Agree (4)
- Strongly agree (5)

Q8 Local helpers (like pastors or teachers) and mental health providers work well together in my community

- Strongly disagree (1)
- Disagree (2)
- Not sure (3)
- Agree (4)
- Strongly agree (5)

Q9 In my community, it is easy for people to keep getting support when moving from community help (like church or school) to help from a mental health professional.

- Strongly disagree (1)
- Disagree (2)
- Not sure (3)
- Agree (4)
- Strongly agree (5)

Q10 A long-term partnership between community help (like churches or schools) and mental health providers is possible in my community.

- Strongly disagree (1)
- Disagree (2)
- Not sure (3)
- Agree (4)
- Strongly agree (5)

Q11 Combining community support (like churches or schools) with online professional mental health services would improve access in my community.

- Strongly disagree (1)
- Disagree (2)
- Not sure (3)
- Agree (4)
- Strongly agree (5)

Q12 In my community, partnerships between community groups (like churches or schools) and mental health providers would be useful.

- Strongly disagree (1)
- Disagree (2)
- Not sure (3)
- Agree (4)
- Strongly agree (5)

Q13 What is the biggest challenge to getting mental health care in your community?

_______________________________________________________________

________________________________________________________________

________________________________________________________________

Q14 What would help community groups (like churches or schools) and mental health providers work better together?

________________________________________________________________

________________________________________________________________

________________________________________________________________

Q15 Which of the following best describes your role?

- Faith leader (pastor, clergy) (1)
- Mental health provider (counselor, therapist, social worker) (2)
- Educator (teacher; teacher's aid) (3)
- Health Care (doctor, nurse, nurse's aid) (4)
- Other (please tell us) (5) __________________________________________________

Q16 What is your gender?

- Male (1)
- Female (2)
- Non-binary / third gender (3)
- Prefer not to say (4)

Q17 What is your highest level of education?

- High school diploma/GED (1)
- Some college (2)
- Associate's degree (3)
- Bachelor's degree (4)
- Graduate degree (5)

Q20 What is your age range?

- 18 - 27 years old (1)
- 28 - 37 years old (2)
- 38 - 47 years old (3)
- 48 - 57 years old (4)
- 58 - 67 years old (5)
- 68 years and older (6)

Q18 How would you describe your community?

- Rural (1)
- Suburban (2)
- Urban (3)
