# Supplementary material for: Using the Hybrid Community Care Model to Examine Implementation Domains in Rural Behavioral Health: Exploratory Pilot Study
Source: JMIR Form Res. 2026 Aug 25;10:e102681. doi: 10.2196/102681 (PMC13505968; doi:10.2196/102681)
Supplement: Supplementary file 2 [file formative-v10-e102681-s002.docx]

**Checklist for Reporting Results of Internet E-Surveys (CHERRIES)**

**Manuscript Title**: Examining Implementation Domains in Hybrid Rural Behavioral Health Systems: An Exploratory Pilot Study Informed by the Hybrid Community Care Model

| **CHERRIES Item** | **Response** | **Manuscript Section** |
| --- | --- | --- |
| **Survey design** | Exploratory cross-sectional web-based survey administered using Qualtrics during and immediately following a professional development workshop for rural community stakeholders. | Study Design; Data Collection |
| **IRB approval** | Approved as exempt by the Indiana University Institutional Review Board (no protocol number was required). | Ethical Considerations |
| **Informed consent** | Participants viewed an electronic informed consent statement describing the purpose of the study, voluntary participation, anonymity, and investigator information before beginning the survey. Submission of the completed survey indicated informed consent. | Ethical Considerations |
| **Data protection** | The survey was anonymous. No personally identifying information was collected. Responses were stored securely within Qualtrics before export for analysis. | Ethical Considerations |
| **Survey development** | Survey items were developed from the Hybrid Community Care Model (HCCM) and supporting literature on rural behavioral health, digital health implementation, care coordination, and integrated care. Because no validated instrument existed, the survey represents an initial operationalization of the implementation domains. | Survey Development |
| **Development and testing** | The electronic survey underwent technical pretesting prior to administration to verify item functionality, navigation, and electronic data capture within Qualtrics | Survey Development |
| **Open vs closed survey** | Closed survey available only to registered workshop attendees through a Qualtrics survey link accessed by QR code or follow-up email. | Participants and Recruitment; Data Collection |
| **Contact mode** | Participants were first informed about the survey during the workshop. | Data Collection |
| **Advertising** | Workshop facilitators introduced the study, explained its purpose, emphasized voluntary participation, and displayed a QR code linking to the survey. No public advertising occurred. | Data Collection |
| **Web/e-survey** | Administered electronically using Qualtrics XM (Qualtrics LLC, Provo, UT, USA). | Data Collection |
| **Context** | Survey administered during a regional rural professional development workshop attended by professionals and community stakeholders working in behavioral health, education, youth services, public health, and related systems. | Setting, Participants, and Recruitment |
| **Mandatory/voluntary** | Participation was voluntary. | Data Collection; Ethical Considerations |
| **Incentives** | No incentives or compensation were provided. | Ethical Considerations |
| **Dates** | Survey administered during the workshop and remained available electronically for 24 hours afterward. | Data Collection |
| **Randomization of items** | Survey items were presented in a fixed order. | Not applicable |
| **Adaptive questioning** | Not used. | Not applicable |
| **Number of items** | Twelve substantive implementation items plus demographic questions. | Measures |
| **Number of screens** | Single-page Qualtrics survey. | Measures |
| **Completeness check** | Participants could skip any survey item; responses were not forced. | Data Collection |
| **Review step** | Participants could review responses before final submission using default Qualtrics setting. | Not explicitly stated |
| **Unique site visitors** | Eighty-two workshop attendees were invited to participate. | Participants and Recruitment |
| **Participation rate** | Thirty-eight of 82 invited participants completed the survey (46.3%). | Participants and Recruitment |
| **Completion rate** | Thirty-six surveys were completed during the workshop, and two additional surveys were completed within 24 hours after the follow-up email. | Data Collection |
| **Cookies/IP checks** | Qualtrics survey settings restricted participants to a single submission. No duplicate responses were identified during data cleaning. | Data Collection (recommended addition) |
| **Handling incomplete questionnaires** | Participants could skip individual items. Analyses used available paired observations. One response containing no substantive data was excluded prior to analysis. | Data Analysis |
| **Questionnaires submitted with atypical timestamps** | Not evaluated. | Not applicable |
| **Statistical correction** | No weighting or statistical adjustment was applied because this was an exploratory convenience sample. | Data Analysis |
